# Pancreatic Resection for Carcinoma of the Pancreas and the Periampullary Region. A Twenty-Year Experience

**DOI:** 10.1155/1990/35325

**Published:** 1990

**Authors:** M. I. Kairaluoma, M. Ståhlberg, H. Kiviniemi

**Affiliations:** Department of Surgery University of Oulu Oulu SF-90220 Finland

## Abstract

410 patients were treated for pancreatic and periampullary carcinoma in 1968–1987 of whom 89 (21.5%) underwent resection. Hospital mortality decreased from 33% in 1968–1972 to 0% in 1983–1987, but the morbidity rate remained unchanged. The trends were similar in patients ≥ 70 and < 70 years of age. The pylorus-saving technique did not increase mortality, morbidity, operative blood loss or the incidence of delayed gastric emptying, but it did reduce the operative time by one hour (p< 0.01). The real 5 year survival for periampullary cancer was 52%, but none of the patients with pancreatic carcinoma survived for 5 years.

It is concluded that age as such is not a limiting factor for pancreatic resection. Resection can be performed with acceptable mortality and survival rates even in patients over 70 years of age if enough attention is paid to careful patient selection and proper preparation. The long-term prognosis is nevertheless related to tumour histology. The recent decline in operative mortality is mostly due to the resections being performed by the same group of surgeons. The best biopsy, and also palliation, is radical removal of the suspicious mass, provided that this can be performed with minimal risk.


*HPB Surgery*, 1990, Vol. 2, pp. 57–67
Reprints available directly from the publisher
Photocopying permitted by license only

© 1990 Harwood Academic Publishers GmbH
Printed in the United Kingdom

### PANCREATIC RESECTION FOR CARCINOMA OF THE PANCREAS AND THE PERIAMPULLARY REGION. A TWENTY-YEAR EXPERIENCE

M.I. KAIRALUOMA, M. STÅHLBERG and H. KIVINIEMI

*Department of Surgery, University of Oulu, SF–90220 Oulu, Finland.*

410 patients were treated for pancreatic and periampullary carcinoma in 1968–1987 of whom 89 (21.5%) underwent resection. Hospital mortality decreased from 33% in 1968–1972 to 0% in 1983–1987, but the morbidity rate remained unchanged. The trends were similar in patients $\geq 70$ and $< 70$ years of age. The pylorus-saving technique did not increase mortality, morbidity, operative blood loss or the incidence of delayed gastric emptying, but it did reduce the operative time by one hour ($p < 0.01$). The real 5 year survival for periampullary cancer was 52%, but none of the patients with pancreatic carcinoma survived for 5 years.

It is concluded that age as such is not a limiting factor for pancreatic resection. Resection can be performed with acceptable mortality and survival rates even in patients over 70 years of age if enough attention is paid to careful patient selection and proper preparation. The long-term prognosis is nevertheless related to tumour histology. The recent decline in operative mortality is mostly due to the resections being performed by the same group of surgeons. The best biopsy, and also palliation, is radical removal of the suspicious mass, provided that this can be performed with minimal risk.

KEY WORDS: Pancreatic neoplasm, pancreatic resection, geriatric surgery

### INTRODUCTION

Several reports have recently suggested a sharp decrease in mortality and morbidity associated with pancreatic resection[1–3] and emphasize the advisability of the pylorus-preserving Whipple procedure[2,3]. Even an age of 70 years or more is not an absolute contra-indication for pancreatic resection[4]. We review here our 20 year experience of pancreatic resection for pancreatic and periampullary carcinoma to determine whether similar trends are evident.

### PATIENTS AND METHODS

410 patients were treated at Oulu University Central Hospital for carcinoma of the pancreas and the periampullary region during the years 1968– 1987, 89 of whom (21.5%) underwent resection, 192 (46.8%) a palliative bypass procedure and 71 (17.2%) laparotomy and biopsy, while 58 (14.2%) were treated non-operatively. Four additional patients, comprising 4% of the original total of 93 resected tumours,

Correspondence to: M.I. Kairaluoma, M.D. Department of surgery, University of Oulu, SF-90220 OULU, Finland.

underwent the standard Whipple procedure for a suspected malignant tumour without mortality, but subsequent microscopic examinations revealed a benign disease - three cases of chronic pancreatitis and one stone impacted at the ampulla. These were excluded from further consideration.

Fifty-one of the 89 patients (57%) underwent resection for pancreatic adenocarcinoma and 38 (43%) for periampullary carcinoma. 65% (58/89) of the resections were considered radical, including 49% (25/51) of those for pancreatic adenocarcinoma and 87% (33/38) for periampullary cancer. In an effort to document recent trends in mortality and morbidity after pancreatic resection, the cases were divided into four 5 year periods: from 1968 to 1972 (N = 3), from 1973 to 1977 (N = 14), from 1978 to 1982 (N = 33), and from 1983 to 1987 (N = 39). The number of bypass procedures and explorative laparotomies decreased during the 20 year period, the resectability rate remained unchanged and the number of non-operated patients increased substantially (Figure 1).

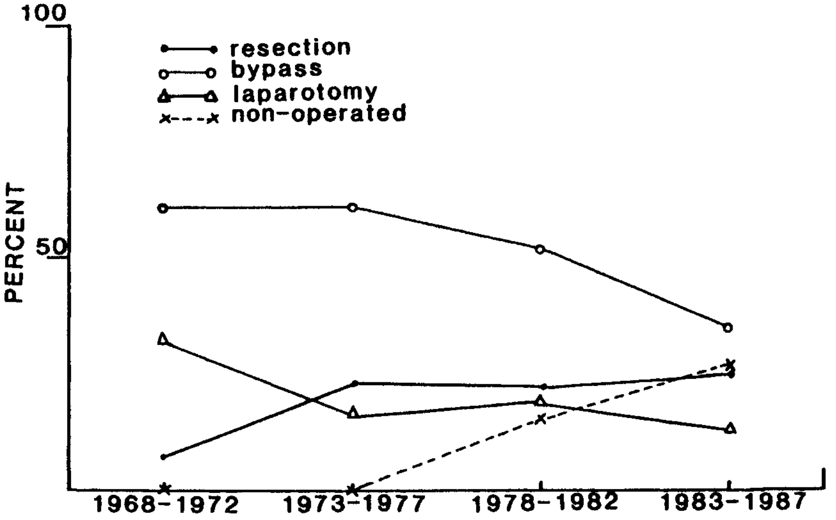

**Figure 1** Treatment.

Twenty-nine of the patients undergoing resection (33%) were aged 70 years or more and 60(67%) were under 70 years of age. A comparison of the groups and types of resection is presented in Tables 1 and 2.

The overall operative mortality rate for pancreatic resection during the earlier years, 1968–1976, was 36%. After that date the operative technique was standardized and the operation was performed regularly by the same surgeons[5]. This retrospective survey also suggests that age as such is not a limiting factor for pancreatic resection, so that all the patients with a resectable tumour who were fit for operation were subjected prospectively to pancreatic resection regardless of age from 1982 onwards. The preferred operation was the standard Whipple procedure. Total pancreatectomy was required only in 10% of these cases, for reasons of radicality. From 1986 onwards, all patients with carcinoma of the head of the pancreas and the periampullary region were subjected prospectively to the pylorus-preserving Whipple procedure if the initial dissection demonstrated no positive nodes or invasion into the portal vein or superior mesenteric vessels.

**Table 1** Comparison of the groups.

| | *No. of patients* | *M/F (%)* | *Age (years) Mean ∓ S.D.* | *Radical resection (%)* | *Adenocarcinoma of the pancreas (%)* |
|---|---|---|---|---|---|
| ≥70 years | 29 | 52/48 | 75∓3 | 62 | 62 |
| <70 years | 60 | 40/60 | 59∓8 | 67 | 57 |
| Total | 89 | 44/56 | 66∓10 | 65 | 58 |

**Table 2** Types of pancreatic resections performed.

| | *Total* | | *Whipple* | | *Distal* | | *Local* | |
|---|---|---|---|---|---|---|---|---|
| | *No.* | *%* | *No.* | *%* | *No.* | *%* | *No.* | *%* |
| ≥70 years | 2 | 7 | 21 | 73 | 5 | 17 | 1 | 3 |
| <70 years | 7 | 12 | 48 | 80 | 5 | 8 | — | — |
| Total | 9 | 10 | 69 | 78 | 10 | 11 | 1 | 1 |

All the patients were followed up until 31 December, 1987, or until death. Seven of the patients aged ≥70 years (24%) and 24 of those aged <70 years (40%) were alive at the end of the observation period, as were 11 of the 49 patients (22%) undergoing resection in 1982 or earlier. All of them had periampullary cancer. None of the 28 patients with pancreatic adenocarcinoma survived for 5 years, the longest surviving patient in this group having died of myocardial infarction 44 months after resection but without any signs of tumour recurrence.

After the exclusion of operative mortality, an actuarial analysis of survival was carried out by the product limit method of Kaplan and Meier. The statistical significance of the differences in survival patterns was determinated by log rank analysis. The difference in operative times between the standard and pylorus-preserving Whipple procedure was analysed using the non-paired, two-tailed Student's t-test. A P-value of <0.05 was chosen as the level of confidence.

### RESULTS

The overall mortality associated with pancreatic resection was 7% and the morbidity rate 37%. Hospital mortality decreased from 33% during the first 5-year period to 0% in 1983–1987, but the morbidity rate remained unchanged (Figure 2). The trends were similar in patients ≥70 and <70 years of age (Figure 3). All the postoperative deaths followed the Whipple procedure, giving a hospital mortality of 9% for this operation. The complication rates for the Whipple procedure, total and distal pancreatectomy, and local excision were 42%, 33%, 10% and 0%, respectively.

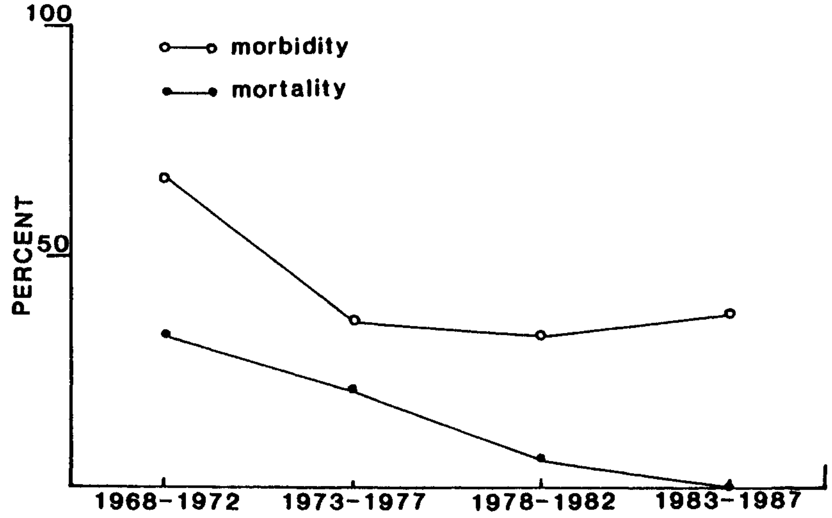

**Figure 2** Mortality and morbidity.

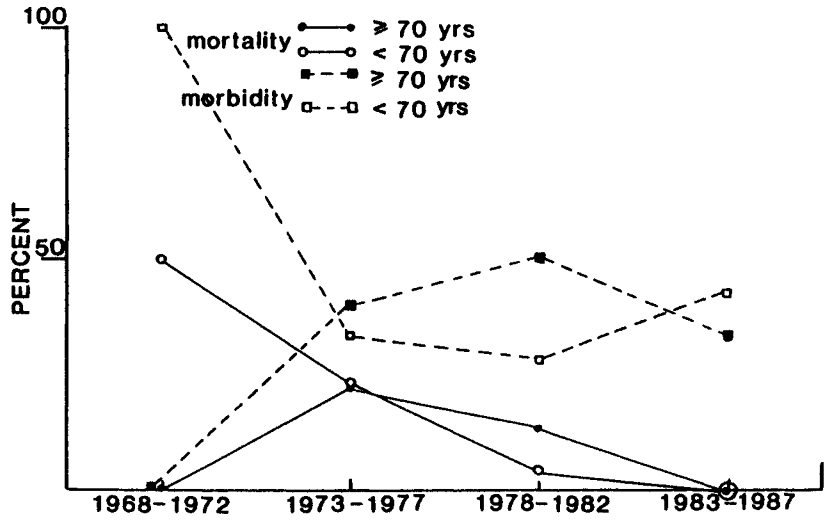

**Figure 3** Mortality and morbidity in patients over and under 70 years of age.

The postoperative complications are analysed in Table 3. Anastomotic leakage occurred in 10 patients. There was no mortality related to biliary leakage, but two of the four leaks from a pancreatic anastomosis proved fatal.

**Table 3** Postoperative complications.

| *Complication* | *No. of patients* | *No. of relaparotomies* | *No. of deaths* |
|---|---|---|---|
| Anastomotic leakage | 10 | 5 | 2 |
| biliary | 6 | 3 | — |
| pancreatic | 4 | 2 | 2 |
| Intra-abdominal sepsis | 11 | 6 | 2 |
| diffuse peritonitis | 3 | 3 | 2 |
| Wound infection | 2 | — | — |
| Major nonsurgical | 12 | — | 2 |
| cardiac | 4 | — | 2 |
| pulmonary | 6 | — | — |
| deep venous thrombosis | 2 | — | — |

A pylorus-preserving technique was used in 12 of the 69 patients undergoing the Whipple procedure (17%). Comparison with the last 12 patients undergoing the standard Whipple procedure (Table 4), showed no hospital mortality in either group. The mean operative blood loss and complication rates were similar in both groups but the pylorus-preserving Whipple procedure reduced the operative time by one hour ($p < 0.01$). By far the most common complication in both groups was transient gastric outlet obstruction, defined as a need for gastric suction for more than 10 days after the operation.

**Table 4** Comparison of the pylorus preserving and standard Whipple procedure.

| | *Whipple procedure* | |
|---|---|---|
| | *Pylorus preserving* | *Standard* |
| Age (mean ∓S.D., years) | 67∓6 | 68∓14 |
| Male/female | 4/8 | 5/7 |
| Operative time (mean∓S.D., min) | 240∓50 | 320∓100* |
| Estimated blood loss (mean∓S.D., ml) | 1500∓700 | 1500∓1300 |
| Hospital mortality (%) | 0 | 0 |
| Morbidity (%) | 25 | 17 |
| Delayed gastric emptying (%)** | 58 | 42 |

* $P<0.01$.
** Gastric suction required for more than 10 days.

Survival is related to tumour histology (Figure 4). There were no 5 year survivors in the pancreatic carcinoma group, whereas the actuarial 5 year survival for periampullary cancer was 46±10% ($p<0.001$). After the exclusion of operative mortality, the real 5 year survival was 52% for periampullary cancer, 40% for ampullary carcinoma, 100% for duodenal carcinoma, and 67% for distal bile duct carcinoma.

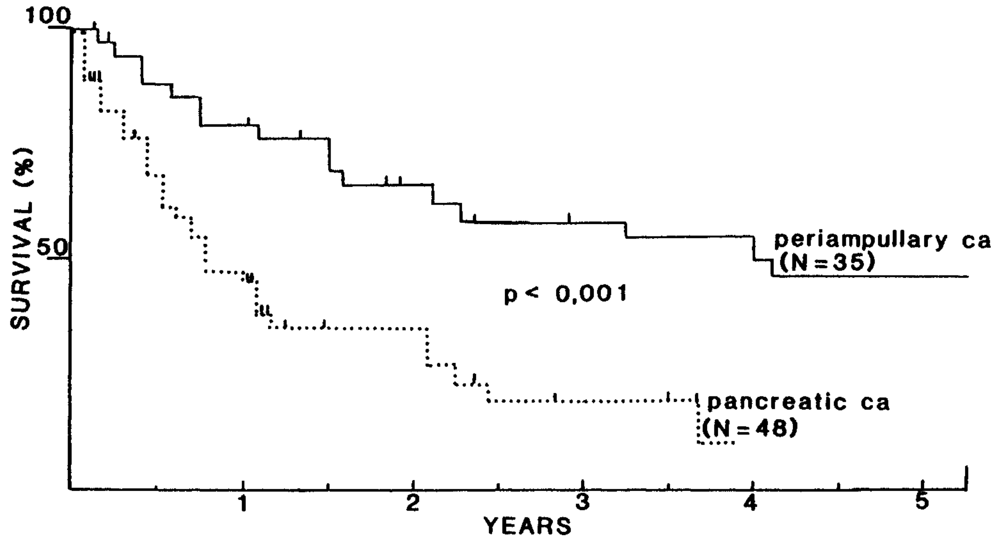

**Figure 4** Kaplan-Meier survival curves for resected periampullary and pancreatic cancer.

Figure 5 shows survival as related to radicality of resection. No patient who underwent palliative resection survived for 3 years. The actuarial 5 year survival for periampullary cancer in patients undergoing radical resection was 50±10%.

Hospital mortality was 7% in the patients ≥70 and <70 years of age and median survival 11 months. The morbidity rate among the patients aged ≥70 years was 38% and that among those aged <70 years 37%. Their actuarial 5 year survival rates were 19 and 37% ($p>0.2$), respectively. The survival curves for pancreatic carcinoma were identical in both groups, but the younger patients fared significantly better ($p<0.025$) after resection for periampullary cancer (Figure 6).

### DISCUSSION

Much of the controversy surrounding pancreatoduodenectomy has focused upon the mortality and morbidity inherent in such an extensive operative procedure. Current reports suggest that mortality is now below 5% and morbidity approximately 20

to 30%[1-3, 6, 7]. The present results clearly reflect this observation, with a substantial improvement especially in mortality, there being no operative mortality among the 39 patients subjected to pancreatic resection during the last 5 year period, 1983–1987. It has also been suggested that the recent decline in operative mortality and morbidity is mostly due to fewer surgeons performing pancreatic resection, strict adherence to a standardized operative technique and the paying of meticulous attention to details in performing the operation. Our results confirm this observation. Since 1976, when we standardized our operative technique and engaged the same surgeons to perform the operation regularly[5], hospital mortality has decreased, having been 33% in 1968–1972 but 0% in 1983–1987. The complication rate remained unchanged, however.

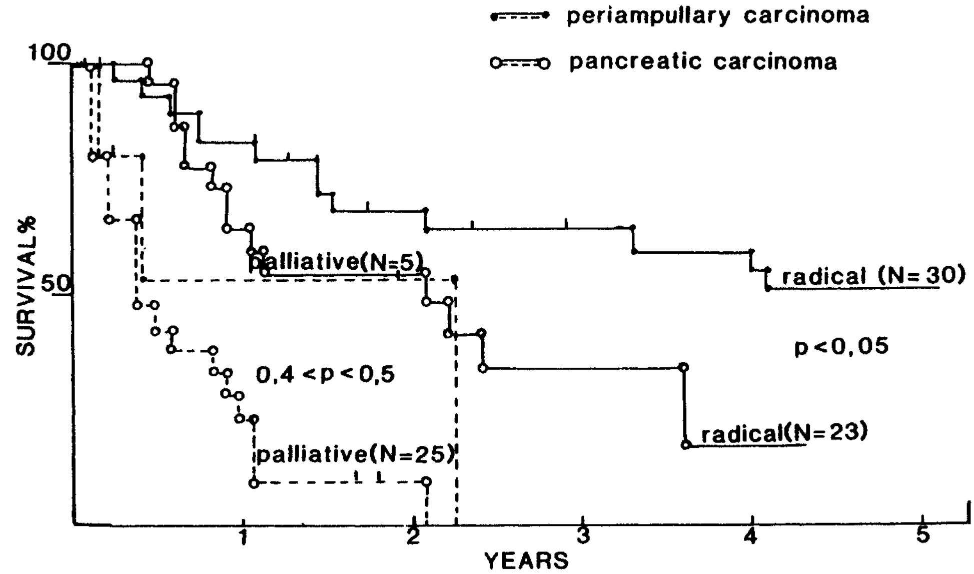

**Figure 5** Kaplan-Meier survival curves for radical and palliative resection.

Most authors emphasize that there is rarely any justification for performing major resection in a patient over 70 years of age owing to prohibitively high mortality and morbidity and a short survival time[8-11]. Our preliminary study suggested that age as such is not a limiting factor for resection[5]. This encouraged the authors to continue an aggressive policy with regard to the treatment of carcinoma of the pancreas and the periampullary region in patients aged 70 years or older. From 1982 onwards, we have prospectively subjected all patients with a resectable tumour who were fit for operation, regardless of age, to pancreatic resection. The overall hospital mortality for pancreatic resection was 7% in both the ≥70 age group and the <70 age group. During the last 5-year period, 1983–1987, mortality was 0% in both groups. Hence pancreatic resection for cancer in the elderly can at present be performed with an acceptable mortality.

It has been suggested, but not clearly demonstrated, that there is no improvement in the prognosis or longevity after pancreatic resection in the elderly, as their average survival time after resection comes close to that for patients undergoing a palliative bypass procedure[8]. In the present series the median and actuarial survival rates were similar in both groups, although the patients with periampullary cancer who were under 70 years of age fared better. The median survival time after resection was twice as long as that reported by us after a palliative bypass procedure[5, 12, 13]. Long-term results were, however, related to tumour histology.

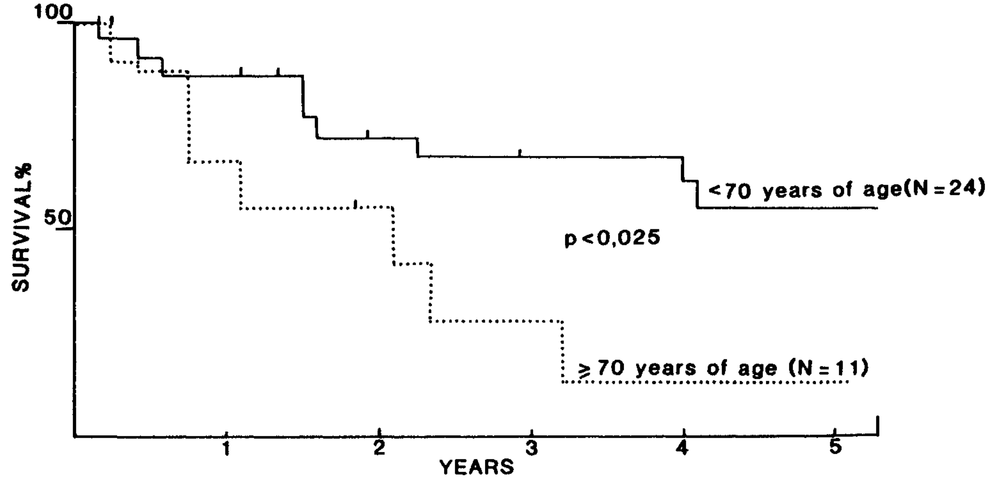

**Figure 6** Kaplan-Meier survival curves for periampullary cancer in patients over and under 70 years of age.

If a tissue diagnosis is not easily accessible or the biopsy results are negative, the question of whether or not to carry out resection on the basis of a strong clinical suspicion of malignancy alone arises relatively frequently during operation. In such a case some authors[1, 14], believe that the best biopsy is radical removal of the suspicious mass, provided that this can be performed with minimal risk. Four patients at this hospital, 4% of the initial total of 93 resected tumours, underwent the Whipple procedure without mortality for a suspicious mass that on subsequent microscopic examination proved to be benign. Hence, we do not necessarily require a tissue diagnosis for pancreatic resection at present.

Although the real 5–year survival rate for periamullary cancer was 52%, none of the patients with pancreatic carcinoma survived for as long as 5 years. Pancreatic carcinoma has usually become a systemic disease by the time of diagnosis and its prognosis is dismal regardless of the mode of treatment. The present survival rates after palliative resection for pancreatic and periampullary carcinoma were similar, the longest survival time in each group being about two years. The question arises of whether this operation should ever be performed as a palliative procedure. The authors are inclined to agree with Trede[1] that if operative mortality is low enough — 3% or less — pancreatic resection is justified even though it may result in palliation for only one or two years.

There was no mortality among the patients undergoing either the standard or pylorus-preserving Whipple procedure, and, as reported earlier by others[2, 3], the complication rate and estimated operative blood loss were similar in both groups. The pylorus-preserving Whipple procedure reduced the operative time significantly, by one hour, since it rendered gastric resection, vagotomy and a large gastrojejunal anastomosis unnecessary[3]. In some studies early postoperative gastric outlet obstruction has not been a problem[2], but it is usually the most common complication[3, 6]. Its incidence can be quite high, up to 50%, and similar with both types of operation, as also in this report. Braasch *et al.*[3] suggest that preservation of the supraduodenal artery is potentially of great importance for ensuring earlier gastric emptying after the pylorus-preserving operation. The present series does not allow any comparison of long-term results, but according to others[2, 3] cancer-free survival after the pylorus-preserving procedure is comparable to that after the standard Whipple procedure.

We conclude that age as such is not a limiting factor for pancreatic resection. Resection can be performed with acceptable mortality and survival rates even in patients over 70 years of age if enough attention is paid to careful patient selection and proper preparation. The recent decline in operative mortality is mostly due to the resections being performed by the same group of surgeons. The long-term prognosis is nevertheless related to tumour histology. The best biopsy, and also palliation, is radical removal of the suspicious mass, provided that this can be performed with minimal operative risk — 3% or less.

### *References*

1. Trede, M. (1985) The surgical treatment of pancreatic carcinoma. *Surgery,* **97**, 28–35.
2. Grace, P.A., Pitt, H.A., Tompkins, R.K., DenBesten, L. and Longmire, W.P., Jr. (1986) Decreased morbidity and mortality after pancreatoduodenectomy. *American Journal of Surgery,* **151**, 141–149.
3. Braasch, J.W., Deziel, D.J., Rossi, R.L., Watkings, E., Jr. and Winter, P. (1986) Pyloric and gastric preserving pancreatic resection. Experience with 87 patients. *Annals of Surgery,* **204**, 411–418.
4. Kairaluoma, M.I., Kiviniemi, H. and Ståhlberg, M. (1987) Pancreatic resection for carcinoma of the pancreas and the periampullary region in patients over 70 years of age. *British Journal of Surgery,* **74**, 116–118.
5. Kairaluoma, M.I., Partio, E., Ståhlberg, M. and Laitinen, S. (1984) Pancreatic and periampullary carcinoma. *Annales Chirurgiae et Gynaecologiae,* **73**, 199–205.
6. Crist, D.W., Sitzmann, J.V. and Cameron, J.L. (1987) Improved hospital morbidity, mortality, and survival after the Whipple procedure. *Annals of Surgery,* **206**, 358–365.
7. Trede, M. and Schwall, G. (1988) The complications of pancreatectomy. *Annals of Surgery,* **207**, 39–47.
8. Forrest, J.F. and Longmire, W.P., Jr. (1979) Carcinoma of the pancreas and periampullary region. A study of 279 patients. *Annals of Surgery,* **189**, 129–138.
9. Herter, F.P., Cooperman, A.M., Ahlborn, T.N. and Antinore, C. (1982) Surgical experience with pancreatic and periampullary cancer. *Annals of Surgery,* **195**, 274–281.

10. Obertop, H., Bruining, A., Eetinck Schattenkerk, M., Eggink, W.F., Jekel, J. and Van Hauten, H. (1982) Operative approach to cancer of the head of the pancreas and the periampullary region. *British Journal of Surgery*, **69**, 573–576.
11. Piorkowski, R.J., Blievernicht, S.W., Lawrence, W.L., Jr., Horsley III, J.S., Neifeld, J.P. and Terz, J.J. (1982) Pancreatic and periampullary carcinoma. Experience with 200 patients over a 12 year period. *American Journal of Surgery*, **143**, 189–193.
12. Kairaluoma, M.I., Myllylä, V., Partio, E., Ståhlberg, M., Laitinen, S., Juvonen, T. and Suramo, I. (1985) Impact of new imaging techniques on survival in cancer of the head of the pancreas and the priampullary region. *Acta Chirurgica Scandinavica*, **151**, 69–72.
13. Kairaluoma, M.I., Kiviniemi, H., Laitinen, S. and Ståhlberg, M. (1987) Staplers in palliative bypass surgery for unresectable pancreatic cancer. *Pancreas*, **2**, 146–151.
14. Jones, B.A., Langer, B.L., Taylor, B.R. and Giotti, M. (1985) Periampullary tumors: which ones should be resected. *American Journal of Surgery*, **149**, 46–52.

### INVITED COMMENTARY

This important paper reports a series of patients undergoing pancreatectomy for cancer at a University Hospital in Finland. The vast majority (78 of 89 patients) underwent resection of the pancreatic head, 9 of these having a total pancreatectomy. The remaining 11 had lesser procedures (all but one distal pancreatectomy), and in my opinion the paper would have given a clearer message if they had been excluded. The overall mortality rate was only 7 per cent; of particular note there were no deaths at all among 39 patients operated during the last 5 years of the study. This figure is particularly creditable when it is appreciated that no less than one third of these patients were over the age of 70 years. The morbidity rate for the operation remains high (30–40 per cent), but morbidity is honestly interpreted to include all but the most trivial of complications.

I find myself in agreement with the authors' philosophy of management for pancreatic and periampullary carcinoma, which can broadly be set out as follows:

1. Resectable cancers of the pancreas should ordinarily be resected, not so much in the hope of cure (which was never achieved in the present series of 52 patients) but because alone among treatments it offers a glimmer of hope; in any case it provides the best palliation. Like Cohen and co-workers[1] I have personally never cured a patient with "ordinary" pancreatic carcinoma, but others have clearly done so[2-4]. A five-year survival rate of 5 per cent can be anticipated after resection for this tumour, though five-year survival alas cannot be equated with cure. The justification for this aggressive policy is an operative mortality rate not exceeding 10 per cent.

2. The survival statistics following resection of "lesser" periampullary cancers (duodenum, bile duct, ampulla, neuroendocrine) are such that resection should certainly be undertaken unless there are clear-cut reasons to the contrary. In fact, patients can expect to live almost as long after resection for ampullary cancer as they can after "curative" resection for colorectal cancer (46 per cent versus 59 per cent five-year survival rates)[5]. For ampullary cancer (only) local excision may do as well or even better than formal pancreatoduodenectomy[6].

3. There is no place for the occasional pancreatectomist. These are high-risk operations in all but expert hands. The authors' operative mortality rate improved dramatically (from 24 per cent to 0 per cent) once they had grasped this point and restricted the operation to those with a special interest. Although pancreatic cancer is on the increase and diagnostic imaging might conceivably allow earlier diagnosis, the overall resectability rate remains low (10–20 per cent), so that the logistics of such a policy are perfectly feasible in most developed countries[7]. Patients with a potentially resectable tumour should be referred to a centre with the appropriate expertise. If a surgeon explores a jaundiced patient for gallstones and finds instead a resectable cancer, I believe he should perform cholecystjejunostomy (or even T-tube drainage) to decompress the biliary tree and then refer the patient if he/she is inexperienced in pancreatectomy.

4. It can sometimes be impossible to distinguish chronic pancreatitis from pancreatic cancer even with the aid of percutaneous or peroperative biopsy. In these cases I heed the advice I received from the late Dr Thomas White of Seattle that the surgeon should undertake "a big biopsy", i.e. a Whipple resection; this is the best treatment for chronic pancreatitis of such severity. It is notable that Dr Kairaloma and his colleagues had four such patients in their series. I am bound to say that the two conditions can usually be separated by the length and nature of the history, the presence or absence of calcification and the operative appearance of the bile duct; sometimes, of course, they will co-exist.

5. There is a very limited role for total pancreatectomy in pancreatic cancer and almost no role for even more radical resections like "regional" pancreatectomy. Preoperative demonstration of portal vein invasion nearly always means that resection is not worthwhile, but xray appearances can be deceptive and once or twice I have resected and reconstituted the portal vein for cancers other than pancreatic carcinoma. I too am persuaded by the advantages of a pylorus-preserving proximal pancreatoduodenectomy (PPPP). In our own experience delayed gastric emptying has only been an occasional problem: 3 of 52 patients required nasogastric intubation beyond 7 days and each settled with conservative treatment[8].

### *References*

1. Cohen J.R., Kuchta, N., Geller, N., Shire, G.T., Dineen, P. (1982) Pancreaticoduodenectomy. A 40-year experience. *Annals of Surgery* **195**, 608–617.
2. Herter, F.P., Cooperman, A.M., Ahiborn, T.N., Antinori, C. (1982) Surgical experience with pancreatic and periampullary cancer. *Annals of Surgery* **195**, 274–281.
3. Tarazi, R.Y., Hermann, R.E., Vogt, D.P., Hoerr, S.O., Esselstyn, C.G. Jr., Cooperman, A.M., Steiger, E., Grundfest, S. (1986) Results of surgical treatment of periampullary tumors: A thirty-five year experience. *Surgery* **100**, 716–723.
4. Trede, M. (1985) The surgical treatment of pancreatic carcinoma. *Surgery* **97**, 28–35.
5. Umpleby, H.C., Bristol, J.B., Rainey, J.B., Williamson, R.C.N. (1984) Survival of 727 patients with single carcinomas of the large bowel. *Diseases of the Colon and Rectum* **27**, 803–810.
6. Knox, R.A., Kingston, R.D. (1986) Carcinoma of the ampulla of Vater. *British Journal of Surgery* **73**, 72–73.
7. Williamson, R.C.N. (1988) Pancreatic cancer: the greatest oncological challenge. *British Medical Journal* **296**, 445–446.
8. Cooper, M.J., Williamson, R.C.N. (1989) Pylorus preservation and gastric emptying during pancreatoduodenectomy. *Gut* **30**, A742.

R.C.N. Williamson
Dept. of Surgery
Royal Postgraduate Medical School
Hammersmith Hospital
London, UK

